# Supplementary material for: Evidence supported by Mendelian randomization: impact on inflammatory factors in knee osteoarthritis
Source: Front Med (Lausanne). 2024 May 28;11:1382836. doi: 10.3389/fmed.2024.1382836 (PMC11165061; doi:10.3389/fmed.2024.1382836)
Supplement: Supplementary file 1 [file Data_Sheet_1.ZIP › data/GCST90274782_5e6/782.forest.pdf]

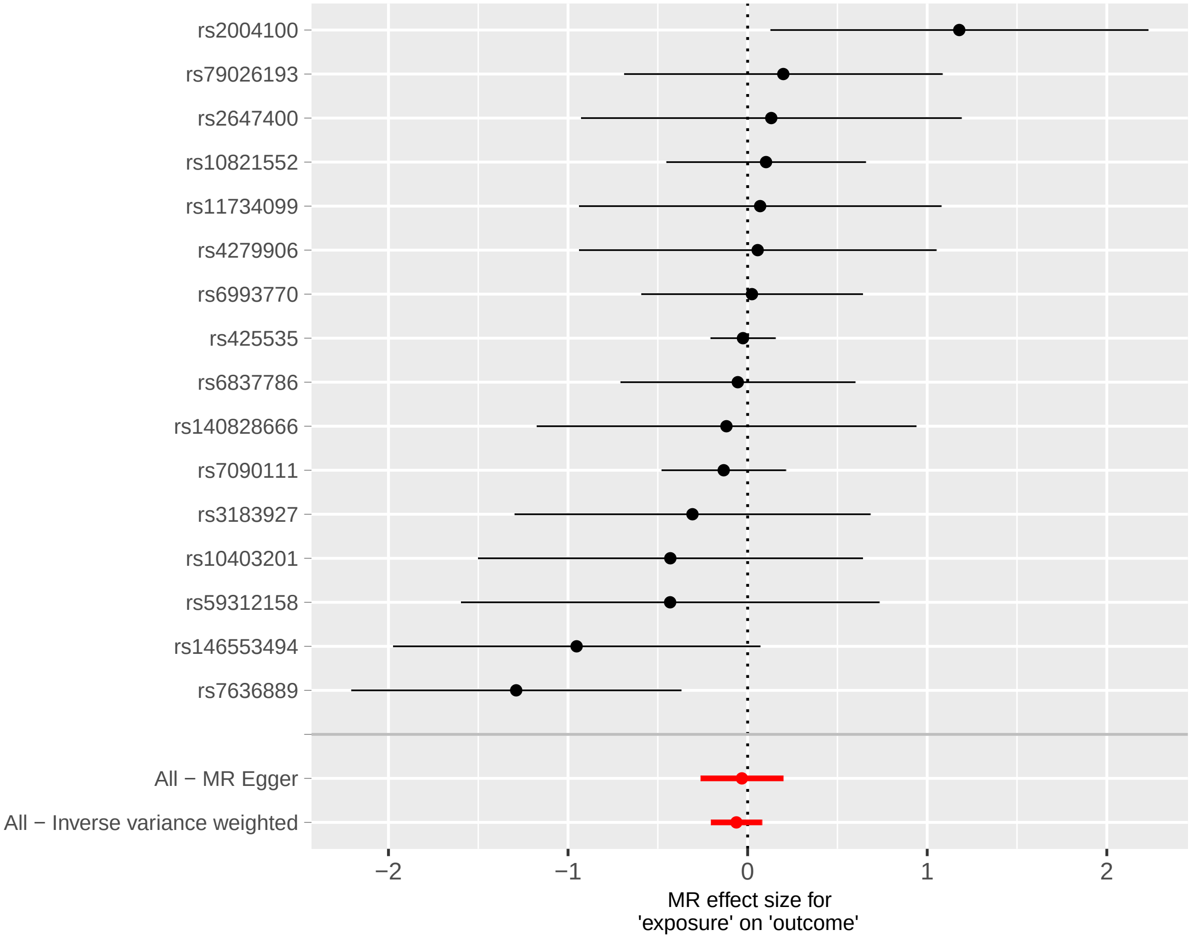
rs2004100
rs79026193
rs2647400
rs10821552
rs11734099
rs4279906
rs6993770
rs425535
rs6837786
rs140828666
rs7090111
rs3183927
rs10403201
rs59312158
rs146553494
rs7636889
All − MR Egger
All − Inverse variance weighted
−2
−1
0
1
2
MR effect size for
'exposure' on 'outcome'
